# Improved harmonisation from policy dialogue? Realist perspectives from Guinea and Chad

**DOI:** 10.1186/s12913-016-1458-7

**Published:** 2016-07-18

**Authors:** Aku Kwamie, Juliet Nabyonga-Orem

**Affiliations:** 1 Ghana Health Service, Research and Development Division, Private Mail Bag, Ministries, Accra, Ghana; 2 Health Systems and Services Cluster, World Health Organization Regional Office for Africa, B.P. 06 Brazzaville, Congo

**Keywords:** Policy dialogue, Harmonisation, Alignment, Guinea, Chad

## Abstract

**Background:**

Harmonisation is a key principle of the Paris Declaration. The Universal Health Coverage (UHC) Partnership, an initiative of the European Union, the Government of Luxembourg and the World Health Organization, supported health policy dialogues between 2012 and 2015 in identified countries in the WHO African Region. The UHC Partnership has amongst its key objectives to strengthen national health policy development. In Guinea and Chad, policy dialogue focused on elaborating the national health plan and other key documents. This study is an analytical reflection inspired by realist evaluative approaches to understand whether policy dialogue led to improved harmonisation amongst health actors in Guinea and Chad, and if so, how and why.

**Methods:**

Interviews were conducted in Guinea and Chad with key informants at the national and sub-national government levels, civil society, and development partners. A review of relevant policy documents and reports was added to data collection to construct a full picture of the policy dialogue process. Context-mechanism-outcome configurations were used as the realist framework to guide the analysis on how participants’ understanding of what policy dialogue was and the way the policy dialogue process unfolded led to improved harmonisation.

**Results:**

Improved harmonisation as a result of policy dialogue was perceived to be stronger in Guinea than in Chad. While in both countries the participants held a shared view of what policy dialogue was and what it could achieve, and both policy dialogue processes were considered to be well implemented (i.e., well-facilitated, evidence-based, participatory, and consisted of recurring meetings and activities), certain contextual factors in Chad tempered the view of harmonisation as having improved. These were the pre-existence of dialogic policy processes that had exposed the actors to the potential that policy dialogue could have; a focus on elaborating provincial level strategies, which gave the sense that the process was more bottom-up; and the perception that there were acute resource constraints, which conditioned partners’ interactions.

**Conclusions:**

Policy dialogue improves harmonisation in terms of fostering information exchange amongst partners; however, it does not appear to influence the operational procedures of the actors. This has implications for aid effectiveness.

## Background

### Harmonisation and policy dialogue

The Paris Declaration outlines the commitments of donor partners and country governments to improve aid effectiveness. Harmonisation, here defined as “harmonised actions, transparency and collective effectiveness; common arrangements and simplified donor procedures; complementary division of labour; and incentivised collaboration” [1], is a key principle of the Paris Declaration. Alignment, another key Paris Declaration principle, is defined as “donor overall support based on partner country national development strategies, institutions and procedures; donor alignment with partner strategies; use of strengthened country systems, and partner countries strengthening their development capacities; public financial management capacities with the support of donors; and untied aid” [1]. Recently, the International Health Partnership (IHP+), a group of donor agencies, governments and civil society organisations committed to operationalising the international principles for development cooperation in the health sector, identified seven areas for action in applying the Paris Declaration principles to health sector development. These are (1) providing well-coordinated technical assistance, (2) supporting South–South and triangular cooperation, (3) using one information and accountability platform, (4) harmonising with and aligning to national procurement and supply systems, (5) harmonising and aligning national financial management systems, (6) recording all funds for health in the national budget, and (7) supporting a single national health strategy [2].

In 2012 the Government of Luxembourg entered into a partnership with the World Health Organization (WHO) to build country capacities in 13 countries in the WHO African Region to achieve health sector results, working towards universal health coverage (UHC). This was in line with harmonisation of health aid in Europe and part of a larger European Union-WHO partnership (referred to as the UHC Partnership) to strengthen national health policy development and, where appropriate, aid effectiveness. The emphasis of the UHC Partnership was to improve the policy dialogue processes in countries of focus through the specific objectives of supporting the development and implementation of robust national health policies, strategies and plans; improving technical and institutional capacities, knowledge and information for health systems; and ensuring that international and national stakeholders’ actions were increasingly aligned with national health policies, strategies and plans, and adhered to other aid effectiveness principles.

While there is no universal understanding of what policy dialogue is, it is broadly considered to be a process of decision-making integrated into the policy-making process to contribute to the change of a policy or development of one, based on evidence-based discussion, workshop interaction and consultation [3]. Policy dialogue may have diverse outcomes, but it has as a fundamental goal to inform policy. Policy dialogue as a process has multiple meanings and functions, and has been variously referred to as “deliberative dialogue” [4, 5], “negotiation over allocation of values” [6] and “interaction between government and non-government organisations to exchange knowledge and experience for development of public policies” [7]. Policy dialogue has been mainly applied in high income settings as a process of public policy development. Increasingly, since the Paris Declaration, policy dialogue has been used as a way of supporting harmonisation and alignment between donors and recipient country governments. Studies on knowledge translation platforms in both high and low income countries give insights into some of the practical elements of the consultative exchange process [8, 9]. The literature on policy dialogue, particularly in low and middle income country health systems, though, is lean (Nabyonga-Orem et al., this issue). Policy dialogue is indicated to boost trust, accountability, transparency, buy-in and ownership for policy decisions [3]. In other words, policy dialogue can contribute to improved health sector governance and ultimately better health policy. Greater contextualised understanding is needed to explain why or why not health policy dialogue works (i.e., brings about its observed outcomes), for whom and under what conditions. The research questions underpinning this study, therefore, seek to find out: does policy dialogue lead to improved harmonisation amongst health actors, and if so, how and why? The study investigates these questions using two countries of the UHC Partnership: Guinea and Chad.

### Policy dialogue in Guinea and Chad

**Fig. 1:**
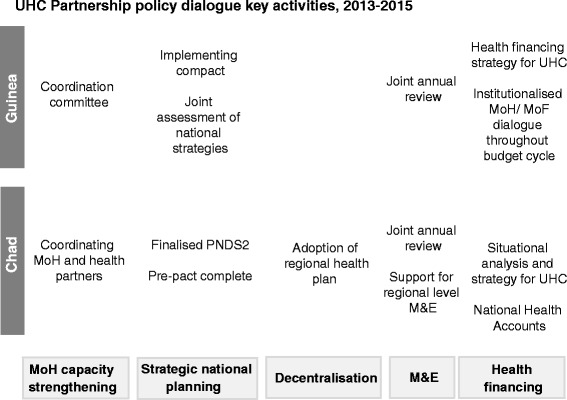
UHC Partnership policy dialogue key activities, 2013–2015

From the beginning of its work in Guinea and Chad, the UHC Partnership set out to support policy dialogue on national health policies, strategies and plans. Identified activities were based on existing policy and planning processes within each country and were designed to fit within the countries’ political contexts and targets. The broad suite of activities was the same across all the UHC Partnership countries, but each country developed its own road map. In Guinea, attention was placed on the key policy documents and strategic planning – including the review of the national health policy 2014, development of a new national health development plan (PNDS 2015–2024), development of the health system recovery and resilience plan (2015–2017) – and the establishment of mechanisms to ensure the effective implementation of the PNDS, including partner coordination meetings. In Chad, the policy dialogue process supported the second PNDS (PNDS2) and provincial health development plans. Activities were elaborated through a series of multi-stakeholder consultations, workshops and technical working groups involving donors, national, regional and district ministry of health (MoH) agencies and relevant civil society actors. The key activities pertaining to the policy dialogue process in Guinea and Chad are outlined in Fig. 1.

Guinea, in West Africa, and Chad, in Central Africa, both have a history of socio-political and economic difficulties and poor health indicators. Guinea has a population of nearly 12 million. In 2013 its under-five mortality rate was estimated at 101 deaths per 1,000 live births, and maternal mortality at 650 deaths per 100,000 live births [10]. Its per capita total expenditure on health is US$ 25. In 2014 Guinea was faced with the challenge of managing an outbreak of the Ebola virus disease.

The population of Chad is 13 million. Its under-five mortality rate is estimated at 148 deaths per 1,000 live births and maternal mortality at 980 deaths per 100,000 live births. Its per capita total expenditure on health is US$ 16. Like many countries in the Region, Guinea and Chad have health systems with pyramidal organisational structures. Both countries are signatories to the IHP+ Compact.

## Methods

### Analytical framework: applying a realist lens

Research has called for more use of realist approaches in evaluating policy dialogue processes [5]. Realist evaluation seeks to draw explanatory causal links among interventions (i.e. policies and programmes), the contexts in which those interventions are introduced, and how those contexts trigger or do not trigger the mechanisms within the interventions that bring about the observed change (i.e. its programme theory or theory of change) [11]. Realist approaches can be useful in assessing large-scale, complex health system interventions [12], and increasingly they are being applied to understand health system transformation in Africa [13–16].

This study is not a realist evaluation. We did not begin with an elaboration of a middle range theory hypothesising how policy dialogue might lead to improved harmonisation to be subsequently “tested” in the case. However, we were inspired to use realist evaluative approaches to guide our data analysis. In this study we used the realist framework of context-mechanism-outcome as our analytical framework. We understood policy dialogue to be the intervention in which context-dependent triggers activate hidden mechanisms to bring about the outcome of interest, in this case improved harmonisation. Here, improved harmonisation was defined as improved alignment of stakeholders to one plan and improved harmonisation of partners. This definition was based on one of the goals of the overall UHC Partnership; that of strengthening aid effectiveness. An initial look at the data confirmed this to be a viable outcome for this study. We defined the context as being both the (1) macro-contexts of country dynamics and the health sector policy and systems conditions into which the policy dialogue process was being introduced, and (2) the micro-contexts of what participants understood the policy dialogue process to be, i.e. their internal perceptions after having engaged with the process. This division of the context into external and internal experiences was important, given that policy dialogue is an inherently people-centred exchange, and, as such, perceptions of what it can achieve will be influenced by how it is understood by the actors involved. Usually, interventions’ mechanisms are hidden [17], so we sought to uncover them through our analysis. To our knowledge no programme theory has yet been elaborated on how policy dialogue brings about change.

### Country selection

Of the 13 countries in the UHC Partnership, Guinea and Chad were purposively selected for this study. This was based on the in-depth familiarity with the policy dialogue experience in the two countries and availability of data. The selection of Guinea and Chad allowed for comparison of two countries that were similar in their outcomes yet had slightly different contexts. This approach supported the accumulation of new evidence, which is needed to answer our research questions. Country-specific, structured interview guides were developed by a team of independent researchers at the WHO Regional Office for Africa. These were forwarded to the WHO Guinea and Chad offices for adaptation and validation. Country-based research teams conducted the interviews, which were face to face.

### Study sampling

The population of interest was the key actors who had been involved in the health policy dialogue processes in Guinea and Chad. These included director generals and directors of service at the MoH, sub-national directors of health, development partners and civil society actors. In Guinea, 28 key informant interviews were conducted. Of the informants, 15 were from the national level of MoH, 4 were subnational directors, 3 were from civil society and 6 represented the development partners European Union, United Nations Children’s Fund, United Nations Population Fund, United States Agency for International Development, WHO and World Bank. In Chad, 14 key informant interviews were conducted involving 6 representatives from the national level of MoH, 4 sub-national directors and 4 development partners. While civil society participated in the policy dialogue, their four key informants were unavailable for interviews because they were on leave.

### Data collection

The interviews were conducted in French from June to September 2015. They lasted between 45 and 60 minutes and were administered at the informants’ place of work. Interview guides were developed aiming to understand the policy dialogue’s deliberation process and outcomes, and participants’ attitudes towards the process. Interview guides were pretested with technical officers from WHO (other country) and revised according prior to data collection. Country documents, including annual reports from the policy dialogues of 2013 and 2014, preliminary data reports and policy documents were collected for review.

### Data analysis

The audio-recorded interviews were transcribed verbatim in French. The data were cleaned and entered into a context-mechanism-outcome matrix for both countries. We worked backwards from the outcomes to figure out the explanations for the mechanisms linking the policy dialogue process to each country context. The data were coded manually in an inductive manner. The manuscript was written in English and the illustrative quotations were translated from French to English.

## Results

### Health policy dialogue implementation in Guinea

The health sector in Guinea has undergone many reforms. The current strategic orientation, which has primary health care as the basis, has sought over time to foster a dynamic of effective governance in the sector to promote transparency and efficient management. To sustain a strategic planning process that is consensual, the coordination structures, which were hitherto insufficient, required re-energising. The policy dialogue process involved (1) conducting a situation analysis; (2) drawing up a national health development plan and initiating its implementation, monitoring and evaluation, and undertaking other actions related to the elaboration of a national compact (i.e. written commitments between the MoH and donor partners outlining their collaboration to achieve health improvements); and (3) carrying out other strategic planning actions at the national and sub-national levels. The onset of the Ebola virus disease in Guinea revealed weaknesses in the coordination of the policy dialogue process at the high levels of the health sector (Nabyonga-Orem et al., this issue).

The respondents largely viewed the improved harmonisation resulting from the policy dialogue process as a correction to the pre-existing fragmentation of the system mainly associated with the participation of multiple actors in the elaboration of the national health plan:

*First of all, the policy dialogue is fully in line with the Paris Declaration on Aid Effectiveness. In regards to alignment, we are currently formulating the health development plan, which will form a basis to align all partners in the same direction. All partners are active in the elaboration of this plan. The alignment focused on taking into account the concerns of all sectoral departments and partners for unanimous and concerted integration into the national policy document*. (National-level MoH informant, Guinea)

Harmonisation was perceived also to be related more to the ability of exchange amongst the partners than to the modification of their procedures. One development partner admitted that:

*True partner harmonisation is not possible because there are numerous procedures in partners’ interventions. In terms of what has improved, we now have many thematic groups within which partners can meet and share knowledge together.* (Donor partner informant, Guinea)

The policy dialogue process was nearly universally understood to be a process of information exchange, as a common platform for prioritising and strategising, to be based on discussion and negotiation, and as having suitability for partner coordination and cooperation. It aimed to align interventions in a single orientation (i.e., it was guided by the national policies), reduce duplication, and advocate for resource mobilisation, integration and maximisation, as expressed by one respondent:

*Several actors operate in the health field according to their own interests. This calls for a dialogue aiming at maintaining priority health objectives. It means carrying everybody along in the same direction. It means rationalising resources and making the system more efficient.* (National-level MoH informant, Guinea)

The respondents were nearly unanimous that the policy dialogue process had been participatory and interactive. The remarks about the way the process unfolded indicate that it gave voice to the participants to freely express their points of view and contribute to the debate in a constructive manner. The deliberation questions were regarded as having been clear. One means by which this was ensured was through the high degree of involvement of the technical working groups in advance of the policy dialogue meetings. The use of data in their preparatory work was viewed as having assisted in the clarity of the objectives of the process, although the participants also applied their own expertise and tacit knowledge in the activities. An informant noted that:

*In terms of how clear the objectives were, concept notes from the thematic working groups were circulated in advance of each technical meeting. The meetings took into account the methodology used and the results achieved. The questions and objectives were clear. However, the dialogue relied more on participants’ opinions than evidence or data.* (National level MoH informant, Guinea)

The respondents also perceived the facilitation of the policy dialogue process as having been well executed. The facilitators were universally referred to as highly skilled in facilitation, knowledgeable of the issues and neutral in their approach to moderating the debate. The participants viewed their counterparts as credible partakers in the dialogue process, because many of them were the actors actually involved in the on-the-ground implementation of the activities associated with the dialogue. No conflicts of interest were perceived by the participants among their colleagues.

There was some dissatisfaction, however, with certain aspects of the policy dialogue process. The informants from civil society and the district level noted that time was not enough to sufficiently debate the issues, and that the process was not sufficiently bottom-up. They noted also that the dialogue should have begun at the local level and built up to develop the national plan, rather than having that plan being driven by the national level.

### Health policy dialogue implementation in Chad

In Chad, the concept of policy dialogue was rather new when the policy dialogue process was introduced in 2013, though previous planning cycles had involved consultative interaction between the MoH and partners from other sectors. The health sector could be characterised as fragmented and lacking in prioritisation. The policy dialogue process was introduced in a context of rapid change in the relationship between the government and other institutions involved in the health actor, such as professional associations and patient groups. The policy dialogue process formalised the consultative dynamic by introducing quarterly meetings with donor partners on the management of the health sector and opening up the planning of the sector, which had been the preserve of managers and professionals. The need for efficient and effective support, plus the dictates of mutual accountability as recommended by the Paris Declaration, had made policy dialogue a new requirement.

The broader context of the policy dialogue process was one of high level political will to improve health outcomes, but leadership within the MoH to coordinate the process was weak, resulting at times to the leading role being assumed by donor partners (issues on coordination challenges of the policy dialogue are covered elsewhere in this issue by Nabyonga-Orem et al.).

The focus of the policy dialogue process was on generating the regional health development plans (PRDS) and PNDS2. The development of the PRDS was launched in 2011 under the direction of the Ministry of Planning, with support from technical committees in charge of developing the PNDS2, and under the leadership of the regional governors. The decentralised units and development partners were also involved in the process. Recently there has been a shift in the relationships of the health actors as a result of the introduction of dialogic processes (which occurred before the UHC Partnership came into being) amongst public sector authorities, non-state actors and development partners to enter into a pre-pact agreement – the signing of which was a major achievement – to leverage the implementation of the first PNDS. The PNDS2 was developed based on previous health plans and the synthesis of 22 regional health development plans. The involvement of other sectors, including the ministries of finance, planning, social action, and public service, was more vigorous in Chad than in Guinea. Relating to harmonisation outcomes, a respondent noted that:

*Although there is a noticeable improvement, it must be said that each partner continues to work with their own procedures. There has been an improvement in terms of information exchange. We exchange a lot of information among ourselves while being fully aware of the distinct set of procedures used by each partner. However, the interventions do not fall outside of the framework outlined by the national health policy in Chad.* (National level MoH informant, Chad)

There were greater concerns in Chad than in Guinea over the ability to implement the subsequent health plans. While the respondents regarded policy dialogue as a collaborative platform, they saw a distinct bilateral quality in the nature of this platform with the Ministry of Public Heath as the other partner. The respondents constantly expressed doubt about the ability of the policy dialogue process to coordinate aid and provide financial and technical assistance to the Ministry of Public Health. Linked to this was the repeated concern about financial and human resource constraints, which heighten the government’s need for such support. According to one respondent:

*What I understand by policy dialogue is that it is the mechanism of bringing together donors and recipients – in other words, the government – aiming at harmonising development aid in a manner that is well-stewarded, transparently and accountably.* (National-level MoH informant, Chad)

The policy dialogue process was widely viewed as participatory, inclusive, evidence informed and well facilitated. Concerns about the less-than-optimal participation of sub-national level actors were noted in Chad as in Guinea. These concerns were related to the fact that the national level Ministry of Public Health was able to mobilise resources but the sub-national could not, which limited its participation in the policy dialogue process. However, the process was perceived as being more bottom-up in Chad than in Guinea, partly owing to the fact that in Chad the PRDS had initially been elaborated at the regional level:

*Everyone was involved from beginning to end. We first started with the regions, we developed the PRDS, we held workshops with all the actors and partners all the way up to the PNDS. So it was done in a participatory manner. If you take the PNDS and read it, you will see what I’m talking about.* (Sub-national level informant, Chad)

### Proposing mechanisms

According to the respondents in Guinea, the common vision and synergised action arising from the policy dialogue gave a sense of participation and inclusion to the actors, who came together in recurrent meetings. The opportunity to understand partner interventions and approaches to particular health issues generated shared confidence among them. This sense of participation and inclusion in the policy dialogue was present also in Chad. Repeated meetings fostered commonality, but this was in the frame of understanding the issue rather than in the vision. A common understanding did lead to confidence; however, the quality of that confidence differed from that in Guinea, because it appears to have been related to transparency in the bilateral partnership between development partners and government actors, as one respondent posited:

*Policy dialogue really allows a coming together; it allows exchange between donor partners and the government. For instance, it avoids the duplication in aid that having multiple partners can bring. If we speak of duplication, it’s about trying to avoid gaps, and filling gaps or failures in programme plans that weren’t clear enough. It allows for better understanding of programme support – and when we speak of understanding we are speaking of accountability, all of which can contribute to the harmonisation of procedures that can possibly result in a sectoral approach in the long run.* (National-level MoH informant, Chad)

The resource constraints were perceived by the respondents to be more acute in Chad and seemed to have tempered the interactions. The significance of the financial and human resource constraints emerged strongly in participant responses. This had two effects. First, resource constraints amplified the focus on resource mobilisation through development aid, thereby conditioning the interactions between development partners and government actors, as one respondent noted:

*In Chad, the policy dialogue is of utmost importance because the country suffers from many shortcomings in terms of policy formulation and implementation, but also in terms of qualified human resources. The policy dialogue programme has indeed assisted to support the Ministry financially and especially technically in the elaboration of strategic documents.* (National-level MoH informant, Chad)

Second, it created tension between national and sub-national level actors. Sub-national actors viewed the Ministry of Public Health, not the development partners, as the source of the institutional weaknesses. For example, commenting on the implementation of the PNDS2, one informant noted:

*Talking about resource mobilisation, it is actually difficult to mobilise resources at the regional level because the resources come mainly from the central level. The implementation of the plan is therefore challenging because whether it be human, financial or material resources, it all comes from the central level. We are under the impression that all resources come from the central level, and sometimes they take time to arrive. In addition, resources are often only partially mobilised, resulting in weakened implementation of plans.* (Sub-national level informant, Chad)

The introduction of the pre-pact process meant that the health sector had been engaged in dialogic processes prior to the introduction of the UHC Partnership and that the actors’ perceptions of what the UHC Partnership policy dialogue could achieve had been pre-primed:

*Well before then there had been a type of policy dialogue in the country – I’m thinking of the pre-pact which had been developed since 2012, during which partners and the government came together to initiate development of the PNDS and PRDS.* (Donor partner informant, Chad)

**Fig. 2:**
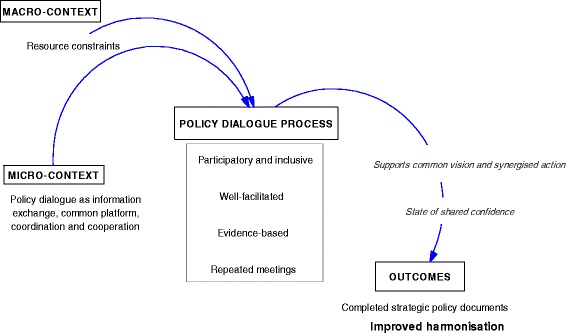
Policy dialogue process in Guinea

**Fig. 3:**
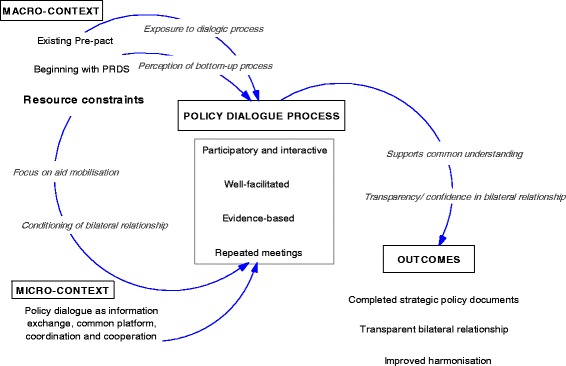
Policy dialogue process in Chad

These contextual differences between Guinea and Chad led to nuanced differences in the perception on the improvement of harmonisation as an outcome of policy dialogue. We illustrate these country-specific policy dialogue pathways in Figs. 2 and 3.

## Discussion

How and why policy dialogue improves harmonisation among health actors are complex. This complexity results partly from the fact that the concept of harmonisation itself is not well defined. While harmonisation simply means to agree or be of one accord, the practice of harmonisation is subject to wide interpretation. The UHC Partnership policy dialogue processes in Guinea and Chad clearly operationalised certain aspects of the Paris Declaration and IHP+ principles such as aligning stakeholder actions around the development of national strategies and increasing transparency. But others, like ensuring that technical assistance was well coordinated, were found to be weak. In Chad, for example, technical assistance arrived late during the policy dialogue process.

Our findings reveal that the participants generally perceived harmonisation to be the degree of participation of the various actors in the strategy elaboration process. While this is one aspect of harmonisation, it is a narrow view. For instance, in Chad, where much emphasis was placed on the management of the bilateral support in the form of the much-needed financial resources and technical assistance, the preoccupation of government actors was on increasing transparency on those resources and their maintained supply. It is noteworthy that the participants were concerned with the ability to harmonise information exchange among actors as opposed to harmonising organisational procedures of actors, which is much more difficult to achieve. We can say that while policy dialogue processes may lead to harmonisation of information from different stakeholders to support sectoral planning, policy dialogue does little for harmonisation of actors’ operational procedures. In Zambia research found that the challenges of harmonising and aligning donors’ organisational procedures with those of the MoH led to diverging perceptions of coordination in the sector [18], highlighting the significant difficulties of increasing country ownership of aid through such processes. Booth [19] points out that strong theories on the relationship between harmonisation and ownership do not exist as yet. It is of significance too that evidence on the Paris Declaration principles has shown that while these principles can enhance aid management and delivery, they are less convincing in yielding sustained reform in policy-making and governance [20]. This raises questions about the reasons such principles are promoted, and about whether policy dialogue can indeed prompt organisational change of agency procedures or whether it is limited to just actor participation and consultation.

It is interesting that in both countries there was a near absence of discussion on the relational aspects among the actors as an aspect of improved harmonisation. A single informant in Chad mentioned the presence of cordiality, motivation and interest in the work to be done as part of the nature of the dialogic relationship. This is important because it suggests that approaches to harmonisation in health sector development are more instrumental, that is focused on aligning stakeholder actions to a strategic plan, than aimed at giving attention to the quality of relationships among the actors. A focus on relational harmonisation would imply the need for a different approach to policy dialogue.

The literature on aid effectiveness demonstrates that there are challenges in maximising effectiveness, including in resource allocation and fungibility of funds, power and information asymmetries, and accountability [21, 22]. In particular, experience from the Sector-wide Approach (SWAp) demonstrates that the phenomena of harmonisation and alignment occur within the broader historical context of the aid effectiveness discourse [23]. Walt and colleagues [24] suggest that over time the idea of aid coordination has transformed into aid management, a non-linear process that can have multiple actors entering and exiting the negotiation process at any time. This may account for our finding that increased participation was one positive outcome of the policy dialogue process. Much of the drive behind aid coordination has emanated from donor partner requirements not from country governments themselves [22], which compounds the challenges of country ownership of aid. This is happening in the context of mushrooming numbers of actors in health and increasing amounts of funding. There is evidence that increases in development assistance have not succeeded in maximising alignment and that sectoral fragmentation, exhibited through the persistence of vertical programming [25, 26], adding another layer of complexity to dialogic process of coordination.

What do our findings suggest about policy dialogue processes in African health systems? The issues relating to transparency, participant commitment, participant mix, dialogue facilitation, preparatory work and mutual understanding that emerge from our data have been shown elsewhere to be important in dialogue processes in the health sector [4]. We found these elements of policy dialogue to be sensitive to the context. In particular, the respondents from both countries largely had a shared understanding of what policy dialogue was, but this understanding was influenced by the broader macro-contexts. For example, while resource constraints were a critical contextual element in both Guinea and Chad, in the Chadian context the degree to which they were a driving element in tempering the bilateral relationship affected the perceptions of harmonisation improvement, even in a situation where the respondents were familiar with dialogic processes and where the process was seen as more bottom-up.

We note some limitations to our study. Time constraints prevented us from conducting a full realist evaluation, which means that our conclusions are limited in their contribution to the building of theory on the conditions and mechanisms that trigger improved harmonisation from policy dialogue. A full realist evaluation would have more completely answered our research questions by exploring other potential mechanisms, such as social networks and stakeholder relationships and power, including historically-driven relationships. This work, however, does contribute to the limited literature on donor engagement and country processes, and lays a foundation for further realist theorising on policy dialogue in African health systems.

## Conclusion

This study finds that policy dialogue processes can lead to improved harmonisation, but this is limited to harmonisation around information generation for sectoral planning, not of actors’ operational procedures to support aid effectiveness. While implementation of policy dialogue was similar in Guinea and Chad and the actors had similar understanding of what policy dialogue is and what it aims for, the broader context – in particular the issue of resource constraints – appears to have influenced the degree of perceived harmonisation. This is related to donor and country interactions, which govern aid management.

## Abbreviations

IHP, International Health Partnership; PNDS, national health development Plan; PRDS, provincial health development plans; UHC, universal health coverage; WHO, World Health Organization
